# A Case of Hospitalization After Pre-operative Interscalene Nerve Block in an Ambulatory Surgery Center

**DOI:** 10.7759/cureus.59717

**Published:** 2024-05-06

**Authors:** Mihir Desai, Conner M Willson, Lyndsey Chitty, Bradley W Gang, Kerri Lydon, Saurin Shah

**Affiliations:** 1 Department of Anesthesiology, University of Florida College of Medicine – Jacksonville, Jacksonville, USA; 2 Department of Clinical Medicine, Des Moines University, West Des Moines, USA

**Keywords:** ultrasound-guided regional anesthesia, phrenic nerve palsy, ambulatory surgical center, diaphragmatic paralysis, interscalene nerve block

## Abstract

Interscalene nerve block (ISB) is an effective and low-risk local anesthetic (LA) procedure that is commonly employed for shoulder surgery. While phrenic nerve involvement occurs to some degree in every ISB procedure, the incidence of hypoxemia and other clinical signs of diaphragmatic disruption is much lower. This is a case of a 36-year-old female with no underlying respiratory disease who developed hypoxemia requiring a night of observation following an ISB for a rotator cuff repair procedure in an ambulatory surgical center. Her hypoxemia was easily treated with supplemental oxygen and she made a full recovery by the next day. The use of ultrasound guidance, reduced LA volume, less potent medication, sterile fluid for optimal visualization, and extrafascial administration should be considered for all patients receiving an ISB to prevent respiratory complications.

## Introduction

Interscalene nerve block (ISB) is a commonly utilized local anesthetic (LA) procedure for post-operative pain control, notably for upper extremity procedures. This procedure involves placing an LA in the interscalene space, an area involving the C5 through C7 nerve roots, located between the anterior and middle interscalene muscles [1-3]. These nerve roots progress distally into the upper and middle trunks of the brachial plexus. This nerve coverage is very suitable for shoulder procedures, as the glenohumeral joint receives innervation from multiple nerves stemming from the C5 and C6 nerve roots [1,4,5]. While a significant portion of cutaneous coverage of the shoulder is achieved through the ISB via the axillary nerve, the remainder of cutaneous innervation is provided by the C3 and C4 nerve roots and is not covered with this block. 

As a result of lying near the anterior surface of the anterior scalene muscle near the C6 nerve root, the phrenic nerve is guaranteed to receive some degree of LA. Prior literature has reported that 100% of patients receiving an ISB will have phrenic nerve involvement [6]. However, the degree to which this is clinically significant is much lower. Most patients receiving an ISB will not experience any clinical signs and symptoms of phrenic nerve palsy, with the exception of certain patient groups [6-8]. This is due to compensation from the contralateral diaphragm and is most likely to occur in patients with adequate pulmonary reserve [9]. This nerve block is relatively contraindicated in patients with significant underlying pulmonary disease, especially chronic obstructive pulmonary disease (COPD), as the ISB has been shown to frequently result in a significant reduction in forced expiratory volume at one second (FEV1) [10-13]. In addition to COPD patients, the block is absolutely contraindicated in patients with a history of contralateral phrenic nerve damage. In high-risk patients, a supraclavicular block is a strong alternative option to reduce the risk of respiratory complications [3,10,11].

While other important anatomical structures such as the jugular veins, common carotid artery, vertebral artery, and pleura lie within close enough proximity to the interscalene space to be at risk for damage, the use of ultrasound guidance helps to avoid these complications [1,14]. Furthermore, inadvertent placement of LA near the T1 nerve root or recurrent laryngeal nerve can result in Horner syndrome or vocal changes, respectively [1]. Given that the effects of LAs are time-limited, any complication related to the inadvertent LA effect is anticipated to resolve within 24 hours of onset. 

Despite these concerns, the ISB is generally considered a safe and effective modality for pain control of the upper extremity. In this case report, we present a rare incidence of complete hemidiaphragmatic paralysis requiring hospitalization in a young patient without contraindications to this procedure. 

## Case presentation

Our patient is a 36-year-old Caucasian female who presented to our ambulatory surgical center for an elective left rotator cuff repair/subacromial decompression procedure. Months prior, the patient had a fall downstairs which resulted in left shoulder dislocation, infraspinatus tendon tear, and a greater tuberosity fracture. She has an underlying past medical history of gastroesophageal reflux disease, Barrett’s esophagus, peptic ulcer disease, hepatic steatosis, and well-controlled mild intermittent asthma. She was overweight with a body mass index of 28. Surgical intervention was delayed in order to accommodate a pending workup for her gastrointestinal pathology. She reported capability to achieve a metabolic equivalent of task level of five.

**Figure 1:**
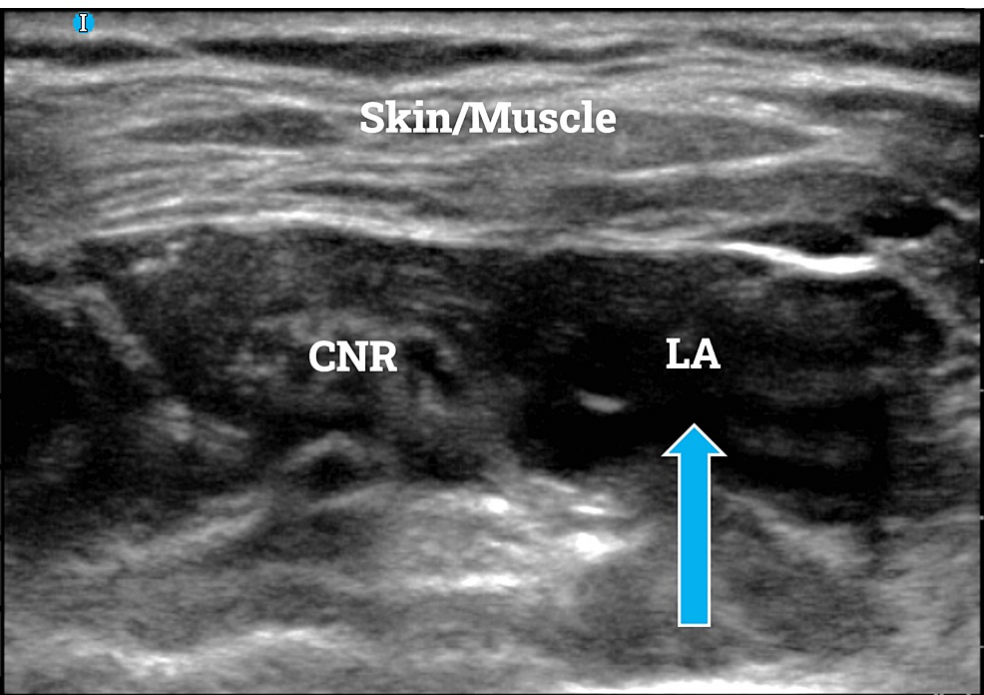
Ultrasound image of interscalene block after administration of 20mL of 0.5% ropivacaine. The blue arrow indicates the local anesthetic surrounding the nerve roots. Due to the patient’s non-traditional anatomy, an increased volume of local anesthetic was utilized. I: indicator; CNR: cervical nerve roots; LA: local anesthetic.

After presentation to our pre-operative unit on the day of her procedure, pain control options were discussed. She consented to receive a single injection of left-sided ISB. This procedure was then set up and completed under ultrasound guidance, utilizing a 2-inch, 22-gauge needle. Upon reaching the perineural space and attempting aspiration, 20 mL of 0.5% ropivacaine (total of 100 mg) was slowly injected into the space while monitoring the patient. An increased volume of LA was utilized, as the patient’s interscalene space was more difficult to characterize on initial ultrasound scans (Figure 1). She tolerated the procedure well and no adverse events occurred. After successful completion of this LA procedure, she was taken to the OR and underwent her scheduled surgery under general anesthesia.

**Figure 2:**
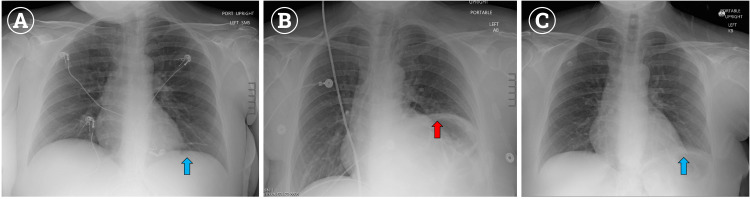
Chest X-ray (CXR) taken nine months prior to the procedure, demonstrating the baseline left diaphragm level (blue arrow) (A); CXR taken in the immediate post-operative period revealing elevated left hemidiaphragm (red arrow), suggestive of left phrenic nerve palsy (B); CXR taken on post-operative day 1 revealing left hemidiaphragm had descended to normal position (blue arrow), indicating resolution of left phrenic nerve palsy (C).

After completion of the surgery and the patient awakening in the post-anesthesia care unit (PACU), an attempt was made to wean her off supplemental oxygen to room air. On room air, the patient desaturated to approximately 70% and would improve to around 95% oxygen saturation once her supplemental oxygen was reintroduced. Multiple attempts yielded similar results over the course of two hours in the PACU. She had reduced breath sounds in the left lung base, but an otherwise unremarkable physical exam. All other vital signs were stable. A chest X-ray (CXR) was ordered and compared to her prior CXR (Figure 2A), revealing an elevated left hemidiaphragm (Figure 2B), but was otherwise unremarkable. These findings were consistent with a unilateral phrenic nerve palsy stemming from her ISB. After discussion with the patient and the surgical team, the decision was made to admit the patient for observation, in order to provide supplemental oxygen and monitor the phrenic nerve palsy until clinical resolution. Upon evaluation the following morning, the patient was maintaining an oxygen saturation of 100% on room air and was otherwise asymptomatic. Repeat CXR revealed that her left hemidiaphragm had descended back to its normal position (Figure 2C). She was discharged home on post-operative day one, with plans for typical outpatient follow up with her surgeon. No complications or other acute issues have occurred since discharge.

## Discussion

This case is unique as it demonstrates a situation in which a young patient with a healthy pulmonary status encountered a complication that significantly altered her post-operative course. While the patient did not experience long-term sequelae or require significant intervention, we believe that it is important to discuss the possible outcomes of a simple procedure like an ISB. 

Phrenic nerve involvement is anticipated to occur in all cases [6], yet most patients tolerate this without intervention [6-8]. As phrenic nerve palsy is a well-known complication of the ISB, multiple variables have been studied to evaluate their effects on this outcome. 

One major consideration is the volume and concentration of the LA utilized in the ISB. In general, lower volume and concentration of the LA are less likely to produce clinical symptoms of phrenic nerve paralysis but are additionally less likely to produce a dense enough block to provide adequate analgesia. Starting with volume, a prior study noted a minimum effective volume for 90% of patients as 8.64 mL of 0.5% ropivacaine [12]. Other previous studies have mentioned utilizing 5 mL of ropivacaine 0.5% or 0.75% as an effective dose for ISB with less likelihood of respiratory complications [8,15]. Another case series determined that the use of low volume of LA in ISB (specifically 5 mL of 0.5% bupivacaine) was effective and less likely to cause respiratory symptoms [16]. In terms of concentration, the use of higher volumes with lower concentrations of LA has not led to less incidence of symptomatic phrenic nerve palsy in multiple studies [17,18]. Conversely, the use of a lower volume but higher concentration of LA has been shown to lead to a greater risk of phrenic nerve paralysis [19]. In our case, a relatively large volume was utilized, which may have led to the development of clinical symptoms in a patient with no known pulmonary disease.

Another consideration is the use of ultrasound guidance, which is generally favored at this point in time compared to landmark techniques with nerve stimulation. Ultrasound has been shown to reduce the number of needle passes and amount of LA required [20,21], which is likely to reduce the risk of phrenic nerve trauma and paralysis, respectively. Furthermore, ultrasound guidance has improved the success rate of ISB [14].

Modifications to the site of LA deposition are another variable that may contribute to the likelihood of symptoms. A prior study found that extrafascial injection of the LA (lateral to the cervical nerve roots) was superior to conventional injection between the C5 and C6 nerve roots in terms of incidence of respiratory complications [22]. Another randomized controlled trial revealed no change in the incidence of phrenic nerve block between anterior and posterior injection methods [13]. This study appears to utilize the same type of conventional injection site (in terms of distance from the nerve roots) across both anterior and posterior approaches, making this distinct from the aforementioned results. In our case, the LA was injected between the nerve roots rather than the extrafascial space, which may have contributed to the severity of this patient’s phrenic nerve palsy.

Another way to reduce the incidence of phrenic nerve paralysis is through utilizing a different regional nerve block that more commonly spares the phrenic nerve. The most frequently used alternative is the suprascapular nerve block, which anesthetizes the suprascapular nerve, comprised of C5 and C6 nerve roots [23]. This block is similar, yet less efficacious compared to an ISB, with reduced risk of respiratory complications [3,24]. Emerging research favors a possible combination of suprascapular nerve block with another upper extremity nerve block, such as an axillary nerve block or infraclavicular nerve block, to provide more comprehensive analgesia while reducing the risk of complications [25,26]. While alternative blocks appear to not be as efficacious when it comes to pain relief in the immediate post-operative period, they tend to reach similar efficacy after this point [26]. Clinicians should choose the most appropriate regional anesthetic plan for patients based on factors such as the patient’s risk factors, the type and extent of shoulder surgery, and the facility at which the procedure is taking place. Lower risk blocks should be heavily considered for all patients at risk of respiratory complications. 

Although precautions with the ISB procedure are typically reserved for those with chronic lung disease, case reports of clinically evident phrenic nerve paralysis have occurred after ISB in otherwise healthy patients [27-29]. Unique to these cases is difficult anatomy requiring multiple injections [23] or the use of very large volumes of LA [28,29]. In our case, the patient had non-traditional interscalene anatomy when examined under ultrasound, which led to using a higher volume of LA for the ISB. For situations like this, sterile fluid can instead be used to hydrodissect and improve visualization of the space, reducing LA burden [30]. Care must be taken to reduce all patients’ risk of respiratory complications related to ISB, regardless of underlying history. Clinicians should consider utilizing an alternative regional pain procedure, such as the supraclavicular nerve block, for patients at high risk of complications [3,10,11].

In our case and all cases studied, significant detriment to patient satisfaction and healthcare resources were evident. Future research that continues to refine and guide techniques for ISB to achieve optimal analgesia with minimal risk of complications will be immensely important for the field of regional anesthesiology. 

## Conclusions

The ISB is an effective procedure for reducing post-operative pain after shoulder surgery. As the phrenic nerve lies adjacent to the interscalene space, patients are at high risk of inadvertent diaphragmatic paralysis. While patients with underlying lung pathology or obesity are at the highest risk of post-operative respiratory symptoms, truly all patients can experience these undesired complications. Physicians should strongly consider utilizing reduced LA volumes, less potent medications, ultrasound guidance, sterile fluid for optimal visualization of nerve structures, and extrafascial administration of LA to balance the lowest risk of respiratory complications with the highest possible analgesic efficacy. 
